# Biomaterial-based sponge for efficient and environmentally sound removal of bacteria from water

**DOI:** 10.1038/s41598-024-61483-8

**Published:** 2024-05-31

**Authors:** Zewang You, Alejandro Lorente, Dini Marlina, Rainer Haag, Olaf Wagner

**Affiliations:** Institute of Chemistry and Biochemistry, Free University of Berlin, Takustr. 3, 14195 Berlin, Germany

**Keywords:** Chemistry, Materials science

## Abstract

Designing materials capable of disinfecting water without releasing harmful by-products is an ongoing challenge. Here, we report a novel polycationic sponge material synthesized from chitosan derivatives and cellulose fibers, exhibiting antibacterial properties. The design of such material is based on three key principles. First, the formation of a highly porous structure through cryogelation for an extensive surface area. Second, the incorporation of cationic quaternary ammonium moieties onto chitosan to enhance bacterial adsorption and antibacterial activity. Lastly, the reinforcement of mechanical properties through integration of cellulose fibers. The presented sponge materials exhibit up to a 4-log (99.99%) reduction within 6 h against both gram-positive *B. subtilis* and gram-negative *E. coli*. Notably, QCHI90/Cell, with the highest surface charge, exhibits a 2–4.5 log reduction within 1 h of incubation time. The eco-friendly synthesis from water and readily available biomaterials, along with cost-effectiveness and simplicity, underscores its versatility and feasibility of upscaling. Together with its outstanding antibacterial activity, this macroporous biomaterial emerges as a promising candidate for water disinfection applications.

## Introduction

For most people, the access to safe water is inadequate. Four billion people, two-thirds of the global population, live under conditions of severe water scarcity for at least 1 month of the year^1^. Water scarcity limits access to clean drinking water and basic hygiene. In these conditions, diseases can proliferate rapidly at home, in schools, or even in health-care facilities. Additionally, antimicrobial resistance from bacteria is expected to be an increasing problem in the near future due to the misuse of antibiotics^2^. It is therefore of high interest to develop new cost-effective strategies and materials that can inactivate bacteria without the use of antibiotics or other chemical substances that are released into the environment.

For water disinfection, chlorination is the most widely used method to kill bacteria, despite its drawbacks^3^. UV irradiation is a more recent method for disinfection. However, besides the need for an electricity source and the cost of acquisition of the equipment, there are limitations with increased distance and beam angles^4^.

Bactericidal additives including metal particles^5,6^, can be integrated into carrier materials like cellulose facilitating passive bacterial adsorption and subsequent inactivation to prevent proliferation and biofilm formation. In the case of active bacteria adsorption via electrostatic interaction of polycationic materials, adsorption and inactivation occur simultaneously as high surface charge materials both bind and kill bacteria^7^. The polycationic surfaces target their cytoplasmatic membrane, which is net-negatively charged. Through this electrostatic interaction, the materials bind the bacterial cells, inhibit proliferation, and can even promote membrane lysis^8^. The advantages of purely polycationic materials over common antibacterial materials or strategies are the absence of leaching chemicals and the independence from an energy source.

Typical polycationic polymers known for their antibacterial properties are chitosan^9,10^, polyethylene imine^11^, and ε-polylysine^12^. These, along with quaternary ammonium groups, have been employed to functionalize other materials like dendrimers^13^, particles^14,15^ graphene derivatives^16–19^, textiles^20^ or hydrogels^21–23^ to incorporate antibacterial properties.

Hydrogels can be prepared as bulk material, films, or cryogels. While films and non-porous hydrogels offer a lower effective contact area, the macroporous structure of cryogels allows bacterial cells to enter the material and adsorb onto their highly increased surface area.

Cryogels are formed via freezing–thawing technique. Generally, a solution of a polymer and a crosslinker is stored at temperatures below the melting point of the solvent (e.g. water, dioxane, or DMSO) which, in its solid state, acts as a pore-forming agent^24^. Once crosslinking is completed, the cryogel is thawed at room temperature and washed with water to remove unreacted residual components/ingredients rendering a porous network with pores surrounded by polymer walls^24–27^. Among the wide variety of materials that can be used to form cryogels, biopolymers have gained great attention during the last decades. In comparison to their fossil fuel counterparts, biopolymers can offer eco-friendly alternatives that are still cost-effective, processable, and offer post-functionalization. Obtained from renewable resources and built up of biodegradable structures, these materials can be included in natural recycling systems.

Cellulose is the most available biopolymer on earth and is used in countless applications. In the context of material science, small contents of cellulose have been demonstrated to improve the structure of cryogels, providing better mechanical properties and performance^28,29^. The addition of 2 wt% of cellulose, for instance, reinforces the mechanical properties of polyimide/CNC hybrid aero-gels significantly^30^.

The structurally related biopolymer chitosan can be produced by extraction of chitin from shrimp shells and other crustaceans and subsequent treatment with alkaline substances. Its amine functionality is the reason for its widely reported antimicrobial properties^31–36^. However, its antimicrobial activity is limited to pH values below 6, when protonation of the amino groups occurs. This can restrict applicability and bioactivity studies in physiological conditions^37^. To enhance its antimicrobial activity and render it independent of pH, permanent cations can be introduced by reacting with glycidyltrimethylammonium chloride (GTMAC). The resulting materials bear quaternary ammonium ions with permanent charge, expected to enhance their antimicrobial activity.

**Figure 1:**
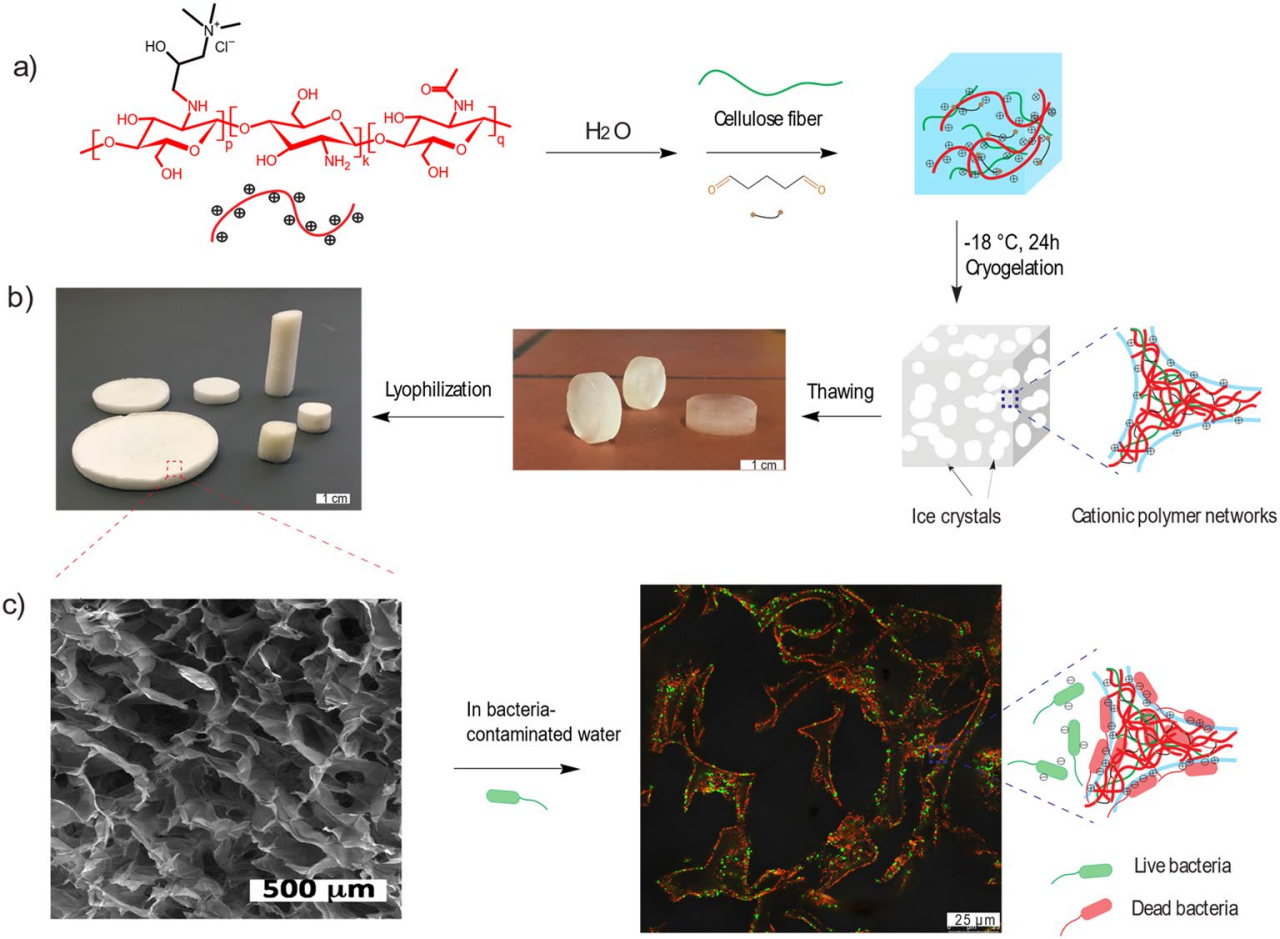
(**a**) Preparation of polycationic sponge materials by cryo-polymerization; (**b**) Image of prepared cryogel sponges in the swollen and dry state; (**c**) scanning electron microscopy (SEM) image of cryogel sponges and fluorescence microscopy image of a live (green)/dead (red) stained *E. coli* incubated chitosan cryogel.

In this work, we present a polycationic sponge material made from chitosan derivatives and cellulose fibers exhibiting antibacterial efficacy against both gram-negative and gram-positive bacteria cells (Fig. 1). The design followed three key principles: (1) implementation of macroporous structure via cryogelation, resulting in high surface area and sponge-like properties, (2) enhancement of bacteria adsorption and antibacterial activity by introducing cationic quaternary ammonium moieties, (3) incorporation of cellulose fibers to reinforce its mechanical properties.

The environmentally sound synthesis utilizing water and abundant biomaterials, offers a low-cost approach suitable for various water purification applications.

## Results and discussion

### Materials design and synthesis

**Scheme 1:**
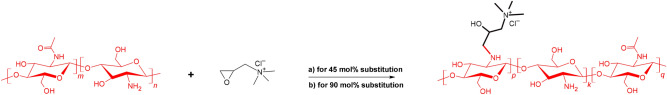
Synthetic scheme for the synthesis of chitosan derivatives. (**a**) **QCHI45:** H_2_O (1% v/v acetic acid), 60 ºC, 18 h. (**b**) **QCHI90:** H_2_O (1% v/v acetic acid), 85 ºC, 6 h.

A series of cryogels and a non-porous chitosan hydrogel (**NPCHI**) were synthesized for comparison purposes. The prepared cryogels consist of pure chitosan (**CHI**), chitosan with 45% and 90% quaternary ammonium group functionalization (**QCHI45** and **QCHI90**, Scheme 1), as well as the same cryogels blended with cellulose fibers (**CHI/Cell**, **QCHI45/Cell**, **QCHI90/Cell**).

Quaternary ammonium group functionalized chitosan was synthesized via epoxide nucleophilic ring-opening reaction with GTMAC. The degree of quaternization (DQ) in chitosan was modified by heating the reaction at different temperatures and times (55 °C/18 h for lower and 85 °C/6 h for higher functionalization degree)^38,39^. The resulting DQ was determined by ^1^H NMR, resulting in 45% and 90 mol%, respectively (Fig. S2).

The prepared chitosan derivatives were used in the same cryo-gelation procedure as pure chitosan. Glutaraldehyde (GA) was selected as the crosslinker for cryo-gelation known for its efficacy with polyamines. The amino groups of chitosan react with two of the aldehyde groups of GA, creating net points of a crosslinked polymer network. Subsequently, the formed Schiff-base imin groups of the network are reduced with sodium borohydride to improve the chemical stability of the cryogels. After cryogel formation, the water was removed via lyophilization. The detailed feed compositions of the series are listed in Table 1, and the detailed synthetic protocols are provided in the supporting information.

**Table 1:** Overview of material composition, mechanical properties, and swelling ratios.

Sample	Chitosan/GA molar ratio	Chitosan/cellulose weight ratio	Degree of swelling [g/g]	Stiffness (storage modulus) [kPa]	Young’s modulus Ec [kPa]
NPCHI	1:1	/	/	/	/
CHI	30:1	/	66 ± 1	3.1	5.1
QCHI45	6:1	/	78 ± 3	1.7	5.7
QCHI90	3:1	/	105 ± 7	1.2	2.1
CHI/Cell	30:1	1:2	20 ± 2	27.3	47.0
QCHI45/Cell	6:1	1:2	22 ± 1	22.5	37.3
QCHI90/Cell	3:1	1:2	23 ± 1	5.3	10.3

### Physical properties

**Figure 2:**
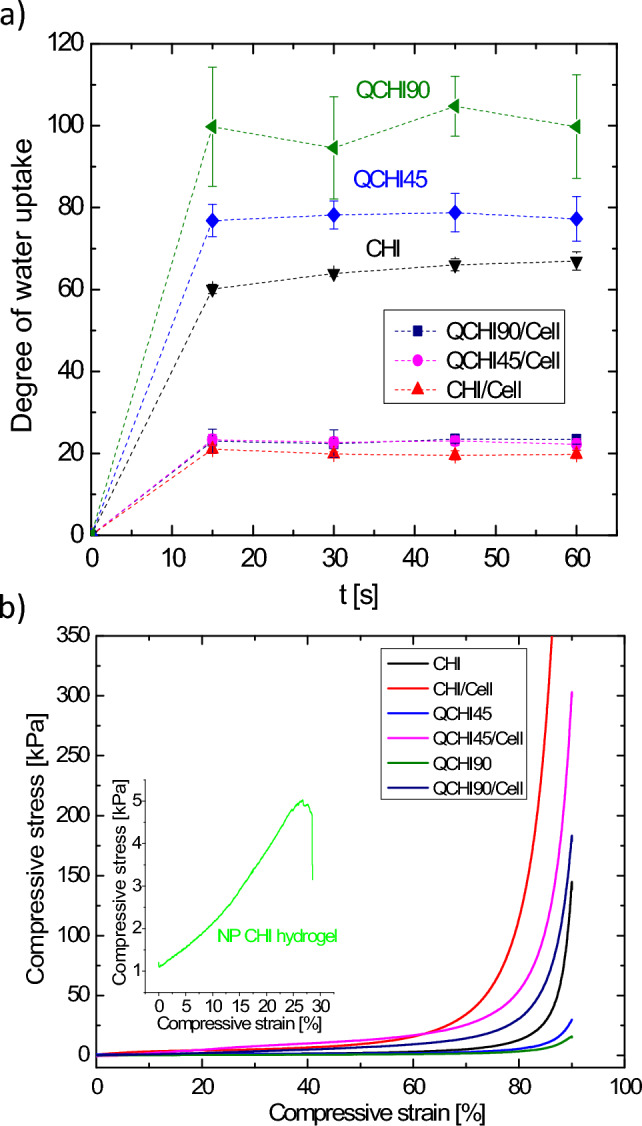
(**a**) Water uptake kinetic of cryogels **CHI, CHI/Cell, QCHI45, QCHI45/Cell, QCHI90,** and **QCHI90/Cell**; (**b**) uniaxial compression tests of non-porous hydrogel **NPCHI**, cryogels **CHI, CHI/Cell, QCHI45, QCHI45/Cell, QCHI90,** and **QCHI90/Cell**.

All synthesized cryogels exhibit an immediate water uptake at contact. This can be attributed to the fast diffusion of liquids into the cryogels due to their high surface-to-volume ratio. The water uptake of the cryogels occurred rapidly within 15 s and reached the swelling equilibrium within 30 s (Table 1 and Fig. 2a). The final degree of swelling expressed as a ratio of weight before and after water uptake, increased with an increasing degree of quaternization from 66 to 105. The hydrophilicity of cryogels was enhanced by introducing quaternary ammonium moieties. As a result, QCHI90, which has a higher degree of quaternization, exhibits the greatest degree of swelling. Conversely, the cellulose fiber reinforced cryogels exhibit a lower water uptake when compared to the chitosan cryogels, with a swelling degree in the range from 20 to 23. The absorbed water can be released again by squeezing the sponge-like materials without damaging the material.

The stiffness of the cryogels was determined from the linear viscoelastic region of the storage modulus. The cryogels that do not contain cellulose in their structure exhibit a lower stiffness in the range from 1.2 to 3.1 kPa than those incorporating it (5.3–27.3 kPa). Furthermore, both classes of cryogels demonstrate an elastic compressive stress–strain behaviour, as illustrated in Fig. 2b. All cryogels can endure significant deformations of up to 90% without becoming damage or permanent deformation. In contrast, the non-porous hydrogel NPCHI experienced mechanical fracture at approximately 24% compressive strain. The tissue-like elasticity of the cryogels, which prevents material damage even at high strains, can be attributed to their interconnected macroporous structure. In addition, the cryogel matrix was strengthened significantly by the incorporation of cellulose fibers, resulting in an increase in Young's modulus from 2.1–5.7 kPa to 10.3–47 kPa. This enhancement makes the cryogel composites more durable and suitable for practical use.

### Cryogel morphology

**Figure 3:**
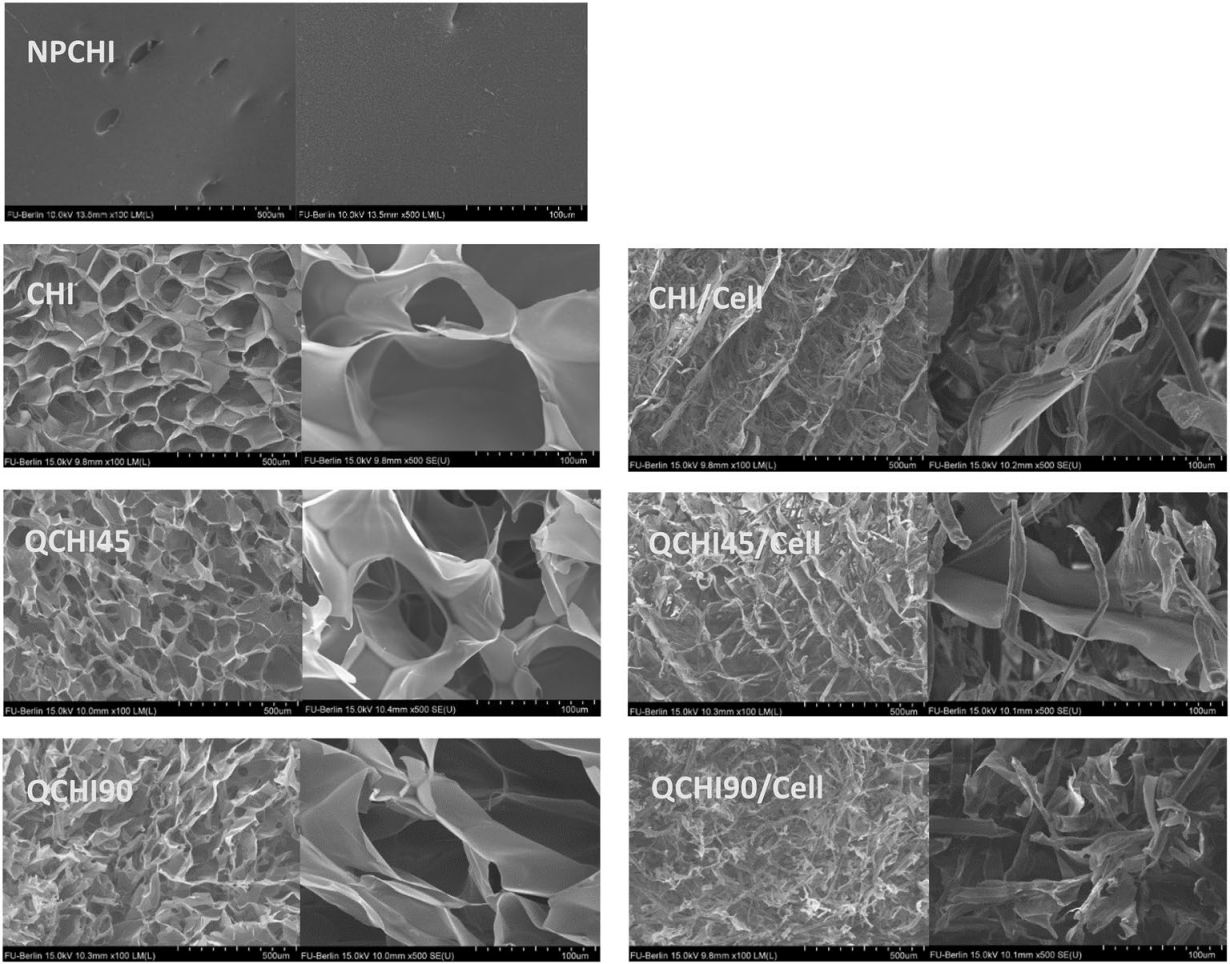
SEM image of NPCHI, **CHI, CHI/Cell, QCHI45, QCHI45/Cell, QCHI90,** and **QCHI90/Cell** at 100 magnifications (left) and 500 magnifications (right), respectively.

The pore morphology of the prepared cryogels and their composites were visualized by scanning electron microscopy (SEM) and a representative image of the physical appearance of NPCHI and CHI/Cell can be seen in Fig. S4. An interconnected highly porous structure can be observed for the **CHI**, **QCHI45**, and **QCHI90** cryogels, confirming the large surface area of the material (Fig. 3). The analysis of pore diameter using ImageJ revealed that the mean pore diameters of the cryogels **CHI**, **QCHI45**, and **QCHI90** were 78 ± 41 µm, 75 ± 38 µm, and 74 ± 41 µm, respectively. Additionally, the distribution of their pore diameters is shown in Fig. S5. In comparison, the cryogels that contain cellulose (**CHI/Cell**, **QCHI45/Cell,** and **QCHI90/Cell**) exhibit a porous structure with smaller pore sizes, and the interpenetration of cellulose fibers through the chitosan walls can be observed.

### Bacteria adsorption studies

**Figure 4:**
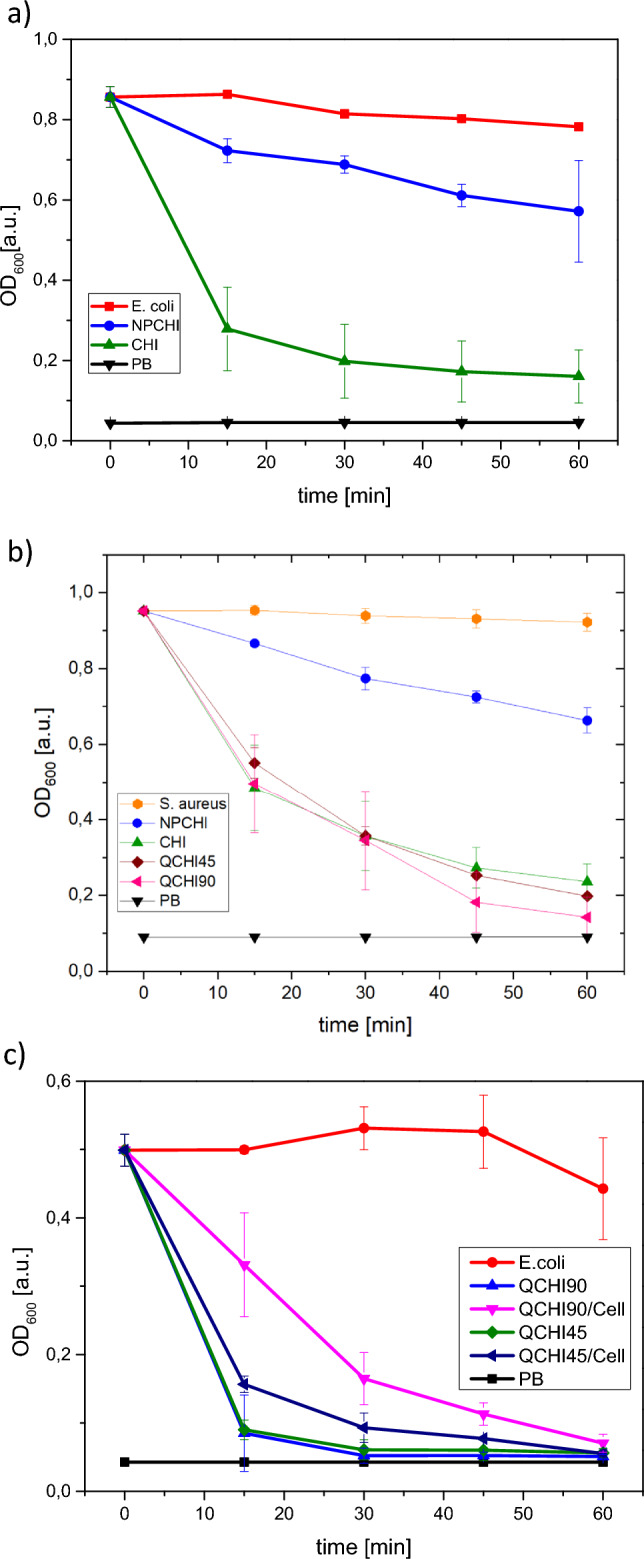
(**a**) Time-dependent OD_600_ measurement of *E. coli* suspensions incubated with **CHI** cryogel and the non-porous hydrogel (**NPCHI**). The PB buffer solution without *E. coli* and an *E. coli* suspension without a sample were used as controls. (**b**) time-dependent OD_600_ measurement of *S. aureus* suspensions incubated with cryogels **CHI**, **QCHI45**, **QCHI90**, and the non-porous hydrogel (**NPCHI**). The PB buffer solution without *E. coli* and a *S. aureus* suspension without a sample were used as controls. (**c**) time-dependent monitoring of OD_600_ values of *E. coli* suspension after incubation with **QCHI45**, **QCHI45/Cell**, **QCHI90**, **QCHI90/Cell**. The control PB buffer solution and the *E. coli* suspension without samples were used as controls.

Investigating the influence of the hydrogels’ porosity on their antibacterial effect, they were first tested against *E. coli* contaminated water via optical density measurement at 600 nm (OD_600_). In multiple paralleled measurements in a 96-well plate, 1 mg of the material was incubated in 0.2 mL of medium for 60 min with a starting OD_600_ value of 0.8, which roughly translates to 10^8^ CFU/mL. In the case of the non-porous hydrogel **NPCHI,** this value decreases to 0.6 after 60 min, which roughly translates to a decrease of 35% of the *E. coli* concentration. In the case of macroporous cryogel **CHI,** the decrease in OD_600_ intensity from 0.8 to 0.2 is observed after 15 min, reaching 0.1 after 60 min, which roughly translates to a decrease of *E. coli* concentration of two orders of magnitude (Fig. 4a). This comparison demonstrates the more efficient adsorption and bacteria removal due to the high surface area and porous structure of the chitosan cryogel.

A gram-positive bacterium *S. aureus* was selected to further investigate the spectrum of bacterial adsorption effect of chitosan cryogels CHI, QCHI45 and QCHI90. Similarly, the OD_600_ values of the residual *S. aureus* suspension were monitored over time as shown in Fig. 4b. For cryogel CHI, the OD_600_ value was reduced from 0.95 to 0.48 after 15 min, reaching 0.23 after 60 min. For the quaternized cryogels QCHI45 and QCHI90, a decrease in OD_600_ from 0.95 to 0.2 and 0.14 respectively was observed after 60 min. The higher efficiency of cryogel QCHI90 for *S. aureus* can be attributed to its higher degree of quaternization. In comparison, the non-porous NPCHI hydrogel showed a decrease in OD_600_ value from 0.95 to 0.66 after 60 min. This further demonstrates that the adsorption efficiency of the cryogels was enhanced due to the presence of the porous structure. The cryogels’ ability to adsorb also gram-positive bacteria *S. aureus* suggests that their bacterial adsorption effect is not specific to one type of bacteria.

To further investigate how the incorporation of cellulose would influence the bacteria adsorption, the *E. coli* adsorption tests were performed with **QCHI45** and **QCHI90** cryogels and their cellulose composites **QCHI45/Cell** and **QCHI90/Cell** similarly. The OD_600_ values of the *E. coli* suspension decreased upon contact with **QCHI45** and **QCHI90** cryogels, dropping from 0.50 to 0.08 after 15 min and further decreasing to 0.05 at 30 min (Fig. 4c). In contrast, the cellulose composites **QCHI45/Cell** and **QCHI90/Cell** exhibit a slower decrease of the OD_600_ value, dropping from 0.50 to 0.15 and 0.33 after 15 min, respectively. Nevertheless, they both reach the same final value around 0.05 after 60 min of incubation. The slower bacteria adsorption rate of the cellulose composites could be attributed to their lower degree of swelling, resulting in a slower diffusion of bacterial cells into the samples. However, the capability as well as the capacity of *E. coli* adsorption is not reduced by the addition of cellulose.

**Figure 5:**
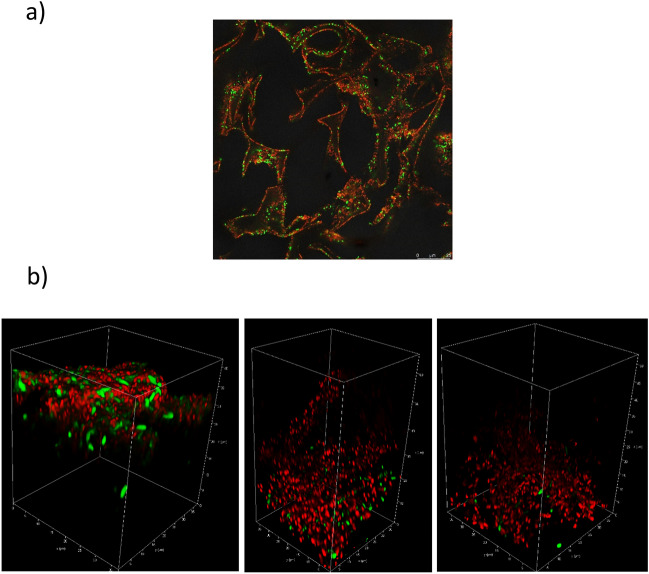
(**a**) Fluorescence microscopy image of a live (green)/dead (red) stained *E. coli* incubated chitosan cryogel (**CHI)**. (**b**) Representative 3D confocal images of cryogels **CHI**, **QCHI45**, and **QCHI90** after loading with bacteria *E. coli* for 24 h (live (green)/dead (red)).

Following the *E. coli* adsorption test, the **CHI** cryogel was immersed in a standard live/dead kit dye solution containing SYTO 9 and propidium iodide to allow the visualization of bacteria by confocal microscopy (Fig. 5a). The interconnected macroporous structure can be observed and the pore walls are covered with live (green) and dead (red) stained bacteria. The ratio of live to dead bacteria after 30 min is roughly 1:5 consistent with the OD_600_ data (Fig. 4a).

Complementary to the previous experiment we aimed to visualize the adsorption and antibacterial activity of **CHI**, **QCHI45,** and **QCHI90**, by incubation for 24 h with live/dead stained *E. coli* cells and imaged via fluorescence microscopy. Due to the interference of cellulose with the dye-staining solution, the other cryogels could not be used. Figure 5b shows the 3D images from multiple recorded z-stacks of the cryogels allowing visualization of the bacteria cells onto the cryogel surfaces. While some of the bacteria cells are still intact and visible in green in the image of **CHI** cryogel, almost all bacterial cells are killed upon contact with **QCHI90** cryogel, in agreement with the increase in quaternary amino groups of the materials.

### Antibacterial activity studies

As the integration of cellulose into the cryogel matrix substantially enhanced the mechanical properties of the cryogels while showing similar adsorption capacities, a quantitative determination of the antibacterial activity of **CHI/Cell**, **CHI45/Cell,** and **CHI90/Cell** was performed.

**Figure 6:**
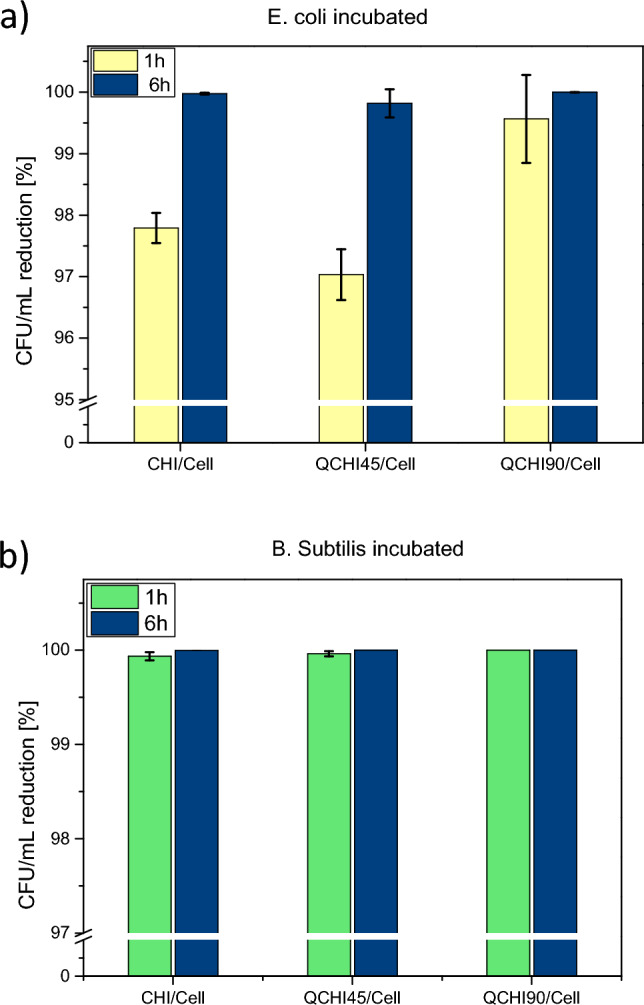
Reduction of colony forming units (CFU) of (**a**) *E. coli* cells and (**b**) *B. subtilis* cells after 1 h and 6 h due to incubation with chitosan-cellulose cryogels with variable degree of amine quaternization.

The cryogels were incubated with gram-negative bacterial cells (*E. coli*) and gram-positive bacterial cells *(B. subtilis).* Briefly, 30 mg of a cryogel sample was incubated with 1 mL bacterial suspension (10^5^ CFU/mL) at 37 °C for either 1 or 6 h. The experimental procedure is described in detail in the supporting information. The reduction of the number of colony-forming units after incubation is shown in Fig. 6.

After 1 h of incubation with *E. coli,* the cryogels showed a 97–99% CFU reduction. Notably, the cryogel with the highest degree of quaternization (**QCHI90/Cell)** decreased the number of *E. coli* CFU by 99.5%, which is a 2-log reduction. This observation suggests a higher rate of antibacterial activity due to the increased number of quaternary amino groups. Upon increasing the incubation time to 6 h, the CFU reduction further increased for all samples to a 3-log reduction and 4-log reduction for **QCHI90/Cell**.

The antibacterial effect on the gram-positive *B. subtilis* was even greater as both **CHI/Cell** and **QCHI45/Cell** showed a 3-log reduction and **QCHI90/Cell** had no detectable viable bacteria corresponding to more than a 4.5-log reduction after 1 h. Similarly, as in the previous case, incubation for 6 h further increase the reduction percentage in all samples, reaching more than 99.99% (4-log) of reduction in CFU for all cryogels.

While both gram-positive and gram-negative bacteria are affected by the polycationic antimicrobial surface of all materials, gram-positive *B. subtilis* seem generally more susceptible possibly due to their thicker peptidoglycan layer or fewer protective structures on their cell surface. Gram-negative bacteria seem to exhibit more resistance due to their outer membrane, which can be overcome by increasing the surface charge density of the materials.

From the material development perspective, the results show that the biomaterial-based **QCHI90/Cell** sponge can disinfect more than 30 times its mass of bacteria-contaminated water within 1 h. Furthermore it exhibits a 4-log reduction over 6 h.

These results highlight the enhanced efficacy of the contact-killing material compared to previously presented polycationic modified chitosan cryogels: 65–95% bacteria reduction within 24 h^31^ or 95–98% within 12 h against *E. coli* and *S. aureus*^32^.

## Conclusions

A series of antibacterial cryogels is presented. Their straightforward syntheses allow for the feasible production of a macroporous biomaterial with a tunable degree of quaternary ammonium functionality and increased structural reinforcement via cellulose fibers. The introduction of cellulose in the cryogel composition reduces the degree of swelling while increasing the strain toughness of the materials.

The antibacterial tests of the presented macroporous chitosan cryogels compared to the non-porous version show the strong effect of increased surface area on the antibacterial effect and indicate that the antibacterial mechanism of the materials is based on surface contact. Interestingly, gram-positive *B. subtilis* cells, which are protected by a thick peptidoglycan layer against physical and chemical stresses, including exposure to antimicrobial agents, are more affected by the antibacterial mechanism of the cryogels than gram-negative *E. coli* cells. Instead of trying to penetrate the peptidoglycan layer, the surface-charged cryogels bind the cell’s surface and exhibit a bacteriostatic effect. However, the live/dead staining fluorescence images also suggest that the adsorbed *E. coli* membranes can be disrupted as the cells show red spots indicating penetrated red dye.

The presented biomaterial-based cryogels exhibit at least a 3-log reduction within 6 h against gram-positive *B. subtilis* and gram-negative *E. coli.* The highest surface charge material **QCHI90/Cell** even exhibits a 2–4.5 log reduction within 1 h of incubation time. This material can disinfect more than 30 times its mass of bacteria-contaminated water within 1 h.

Its antibacterial activity, reinforced matrix, and feasible production render this macroporous biomaterial a promising candidate for applications in water purification systems or medical applications such as wound dressings.

### Supplementary Information

Supplementary Information.

## Data Availability

All data generated during this study are either included in the article and supplementary information, have been uploaded as a supplementary zip file, or are available on request.
