# Supplementary material for: Biomaterial-based sponge for efficient and environmentally sound removal of bacteria from water
Source: Sci Rep. 2024 May 31;14:12496. doi: 10.1038/s41598-024-61483-8 (PMC11143301; doi:10.1038/s41598-024-61483-8)
Supplement: Supplementary file 1 — Supplementary Information. [file 41598_2024_61483_MOESM1_ESM.docx]

Supporting Information

Biomaterial-based sponge for efficient and environmentally sound removal of bacteria from water

Zewang You †*, Alejandro Lorente†, Dini Marlina, Rainer Haag, Olaf Wagner*

Institute of Chemistry and Biochemistry, Free University of Berlin, Takustr. 3, 14195 Berlin, Germany

*Corresponding authors: Dr. Zewang You ([zewangyou@hotmail.com](mailto:zewangyou@hotmail.com)), Dr. Olaf Wagner ([olaf.wagner@fu-berlin.de](mailto:olaf.wagner@fu-berlin.de))

Table of content

[Experimental section 3](#_Toc162647637)

[Materials 3](#_Toc162647638)

[Methods 3](#_Toc162647639)

[Synthesis 6](#_Toc162647640)

[^1^H NMR studies 7](#_Toc162647641)

[IR spectra of chitosan and cryogels 9](#_Toc162647642)

[Comparative images of dried CHI/Cell cryogel and NPCHI 10](#_Toc162647643)

[Distribution of pore diameters of cryogels from SEM images 10](#_Toc162647644)

[Culture plate photos 11](#_Toc162647645)

[References 19](#_Toc162647646)

## Experimental section

### Materials

Chitosan (viscosity 600 mPa.s, 92.7% of deacetylation, 400 kDa) was purchased from Acros and BioLog Heppe GmbH (Landsberg, Germany). Acetic acid, Glutaraldehyde (GA), and Sodium borohydride were purchased from Merck (Darmstadt, Germany). Glycidyl trimethyl ammonium chloride was purchased from Sigma-Aldrich (Taufkirchen, Germany). Solvents were used as purchased unless mentioned otherwise.

Gram-negative stain *Escherichia coli* (*E. coli*, DH5α) and gram-positive stain *Bacillus subtilis* (*B. subtilis*, SH1000) and *Staphylococcus aureus* (*S. aureus*, SH1000)were adopted in this study. The bacteria were initially streaked from -80 °C glycerol stocks on lysogeny broth (LB). After growth on LB agar plates, the cells were cultured from a fresh single colony in LB broth.

LIVE/DEAD BacLight Bacterial Viability Kit for microscopy and quantitative assays (L7012, Molecular Probes, Eugene, OR, USA) was purchased from Thermo Fisher Scientific.

### Methods

**NMR**

An ECX 400 MHz spectrometer (Bruker, Karlsruhe, Germany) was used to record the ^1^H NMR spectra of QCHIs in D_2_O at 25 °C. The NMR spectra of chitosan was measured in D_2_O with the addition of two drops of deuterium chloride 20% solution in D₂O.

**IR**

IR spectra of chitosan and cryogels were collected on a FT/IR-4100 spectrometer (Jasco, Hachioji, Japan) equipped with an Attenuated Total Reflection (ATR) unit.

**OD_600_ determination**

The bacterial cell density was estimated by the turbidity of suspension, which was measured in the microplate reader (Nanotemper, Munich, Germany)

**Water uptake test**

The water uptake capacity (Q) of cryogels was determined by immersing a known mass of cryogel pieces into water. The weight of swollen samples was measured at different intervals to determine the kinetics of water uptake. The Q was then calculated according to equation 1, where m_ex_ is the dry weight of hydrogels and m_sw_ is the mass of the hydrogel swollen in distilled water.

Q= m_sw_ / m_ex_ (1)

**SEM**

Measurements were performed with a field emission scanning electron microscope (FE-SEM, Hitachi SU8030, Japan) at an accelerating voltage of 15 kV, and a current of 10 μA. Briefly, the samples were first lyophilized in a freeze-dryer. When the samples were dried until constant weight was achieved, they were sputtered with 5 nm gold before SEM imaging by using a sputter coater (Emscope SC 500, Quorum Technologies, UK) for 30 s under argon atmosphere at a current of 30 mA and a pressure of 10^-1^Torr (1.3 mbar).

**Confocal microscopy**

The confocal images were taken using a confocal laser scanning platform (TCS SP8, Leica, Germany).

**Rheological** investigations were performed on a KINEX (Marlven, Karlsruhe, Germany) by using an 8 mm plate-plate geometry at 25 °C. The cryogels were first analyzed by amplitude sweeps over a strain range of 0.01% to 10% to determine the linear viscoelastic regions. Frequency sweep measurements of cryogels were performed under a constant strain of 0.1% within the frequency range from 0.01 to 10 Hz. Swollen cryogels in the shape of cylinders were used for the uniaxial compression test. Samples were compressed to 90% of their original height.

All the data were given as mean values ± standard deviation of at least three data points unless mentioned otherwise, and were calculated according to equations 2 and 3. $\overline{A}$ is the mean value, *N* is the number of data points, *V*$\omega$ is the value of the $\omega$^th^ measurement, and *S* is the standard deviation, which describes the spread of the individual values about its mean in a data set.

$$\overline{A} = \frac{1}{N}\sum_{\omega=1}^{N} V\omega\left( 2 \right)$$

$$S= \sqrt{\frac{1}{N-1}\sum_{\omega=1}^{N} \left( V\omega-\overline{A} \right)^{2}} (3)$$

***Bacterial adsorption test***

The bacteria strain of *E. coli* (DH5α) and *S. aureus* (SH1000) was used in the tests. The bacteria were cultivated in the LB agar plates at 37 °C overnight. The bacteria colony was collected and diluted with PB to obtain an optical density at 600 nm (OD_600_) of around 0.8. 2 mg of the cryogel or hydrogel samples or 6 mg of the cryogel cellulose composites were placed in a 96-well plate and then 200 μL bacterial suspension was added. The mixtures were incubated in an incubator (B6060, Heraeus, Germany) at 37 °C. The OD_600_ of bacterial suspension after co-culturing with samples was determined at different time intervals to investigate the adsorption kinetics. Each experiment was repeated three times. After incubation for a certain time, the *E. coli* bacteria-loaded cryogels were stained with SYTO 9 and propidium iodide (Invitrogen) according to the manufacturer’s protocol and subjected to confocal microscopy imaging.

***Antibacterial test***

Overnight cultures were made from transferring a colony (ca. half a loop) from the agar plates to culture tubes containing sterilized Luria-Miller broth (LB, 1% w/v BactoTM Tryptone, 1% w/v NaCl, 0.5% w/v Oxoid Yeast Extract) (~20 mL). Bacterial cultures were incubated overnight at 37 °C with constant shaking speed. The bacteria suspension was centrifuged at 4000 rpm for 5 min. Then the LB was decanted and then PB (pH 7.0) was added to disperse the bacteria particles.

30 mg of the cryogels were placed in 24 well plate centrifuge tubes and then 1 ml bacterial suspension was added. Bacteria suspension without cryogel was used as control. The mixtures were incubated in an incubator (B6060, Heraeus, Germany) at 37 °C for 1 h or 6 h.

50 μl supernatant was pipetted out from the tube at different time intervals and then cultured on the agar plate overnight at 37 °C. On the next day, the number of colonies on the plate was counted.

### Synthesis

***Synthesis of quaternized chitosan derivatives (QCHI):***

QCHI derivatives were synthesized according to the reference.^1^ Briefly, 2 g of chitosan was dispersed in 80 mL of deionized water with 1% (v/v) acetic acid and the solution was stirred for 1 h. The glycidyl trimethylammonium chloride (GTMAC) was then added dropwise into the mixture. The mole ratio of GTMAC to amine groups on the chitosan backbone was 3:1. The reaction mixture was stirred at 55 °C for 18 h or at 85 °C for 6 h to obtain QCHIs with medium and high degree of quaternization. Following the reaction, the undissolved polymer was removed by filtration under reduced pressure, and the filtrate was then precipitated in a pre-cooled acetone/methanol mixture (5:1 v/v). The purified QCHIs were dried under vacuum at 40 °C for 48 h. The DQ of the QCHIs was determined by ^1^H NMR.

***Synthesis of cryogels*:**

Porous hydrogels were synthesized by cryo-polymerization. In general, 2 wt% of chitosan or quaternized chitosan was prepared by dissolving the polymer in deionized water with 1% (v/v) acetic acid. In the case of cryogel composites, a certain amount of cellulose fiber was then added to the solution and mixed homogeneously. The corresponding amount of GA was then added to the pre-cooled chitosan solution. After proper mixing, the mixture was then transferred to molds and placed in a -18 °C refrigerator for 24 h. Afterwards, the samples were thawed at room temperature and extracted with water exchanged every 3 h for at least 6 times to remove unreacted moieties until equilibrium swelling was reached.

## ^1^H NMR studies

**Chitosan**

*Figure S1. ^1^H NMR spectra of* ***chitosan*** *in D_2_O with two drops of deuterium chloride 20% solution in D₂O.*

The degree of acetylation (DA) was determined by the integral ratio of protons in the -CH_3_ group to the H-2, 3, 4, 5, 6, 6’ on the backbone.^2^

DA = (I_CH3_ / 3) / (I_H2-H6_ / 6) * 100 = (4.38 / 3) / (121.22 / 6) * 100 = 7.3%

**Chitosan derivatives QCHI45 and QCHI90**

*Figure S2. ^1^H NMR spectra of* ***QCHI45*** *(top) and* ***QCHI90*** *(bottom) in D_2_O.*

The DQ was determined by the integral ratio of protons in the -N(CH_3_)_3_ group to the H-3,4,5,6,6’ on the backbone.^2^

1. DQ (medium)= (3.91/4.78) : (9/5) * 100% = 45.4%
2. DQ (high)= (7.71/4.76) : (9/5) * 100% = 90%

### IR spectra of chitosan and cryogels


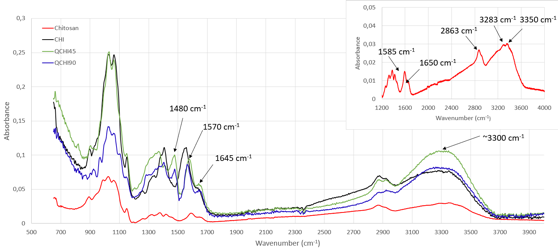


*Figure S3. FT-IR spectra of chitosan, cryogel* ***CHI****,* ***QCHI45****, and* ***QCHI90.***

The characteristic bands of the chitosan were observed at 3283 cm^−1^ and 3350 cm^−1^ (Figure S3), indicating the N-H stretching of primary amines and O-H stretching respectively. The band at 2863 cm^−1^ is due to the C-H symmetric stretching. The bands at 1650 cm^−1^ and 1585 cm^−1^ are attributed to the amide I (C=O) and amide II (N-H), respectively.

All crosslinked cryogels exhibited a broad band at 3300 cm^−1^, indicating the overlap of O-H or N-H stretching vibrations, together with N-H secondary amine stretching of the cross-linked chitosan. Compared to chitosan, the band at 1585 cm^−1^ attributed to the N-H bending of primary amine was not visible for crosslinked cryogels **CHI**, **QCHI45** and **QCHI90**, suggesting a change from primary amine to secondary amine. In addition, a band at 1480 cm^-1^ for both **QCHI45** and **QCHI90**, attributed to the C–H bending of the trimethylammonium group, provides evidence of the introduction of the quarternary ammonium salt group on the chitosan backbone.

### Comparative images of dried CHI/Cell cryogel and NPCHI


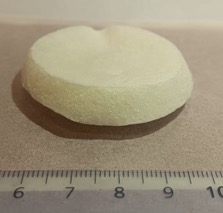

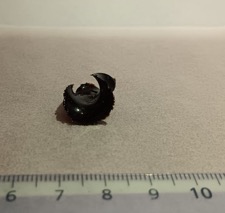


*Figure S4. Photographs of* ***CHI/Cell*** *cryogel (left) and* ***NPCHI*** *(right). As indicative values, the mass of depicted* ***CHI/Cell*** *and* ***NPCHI*** *materials is 0.48 g and 0.30 g, respectively.*

### Distribution of pore diameters of cryogels from SEM images


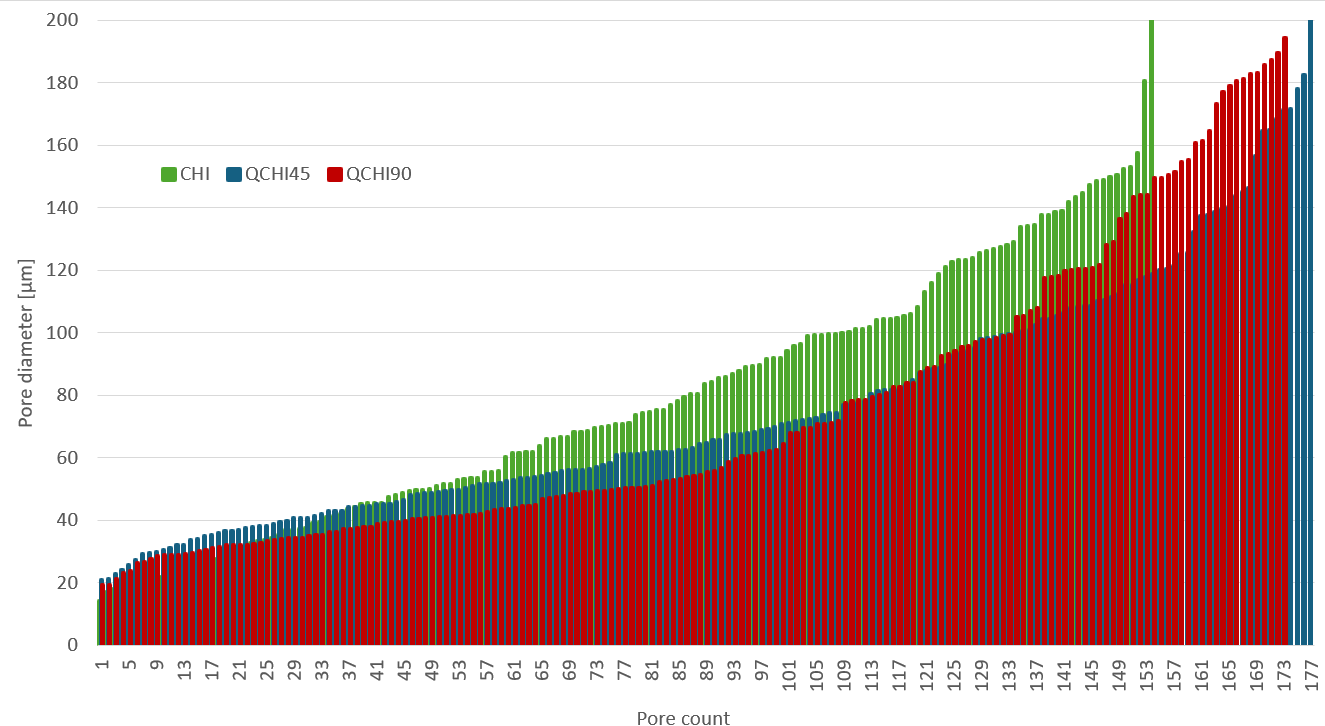


*Figure S5. The analysis of pore diameters of cryogels* ***CHI****,* ***QCHI45****, and* ***QCHI90*** *by using software ImageJ.*

## Culture plate photos

*Fig. S6. All photos of culture plates corresponding to the results shown in Fig. 6 of the main file are shown below.*


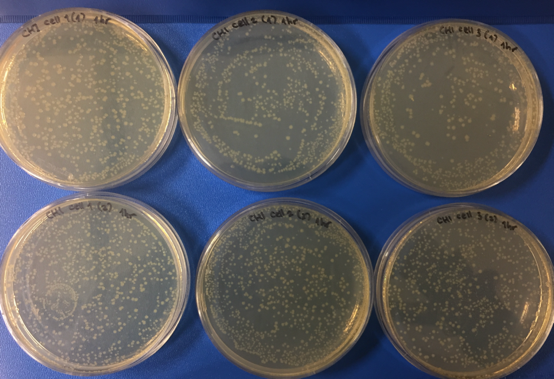


*E. coli* CHI/Cell 1h


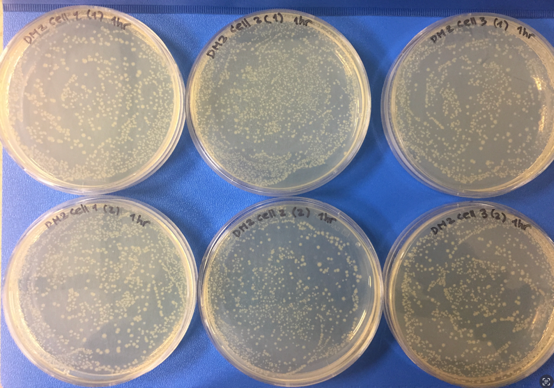


*E. coli* QCHI45/Cell 1h


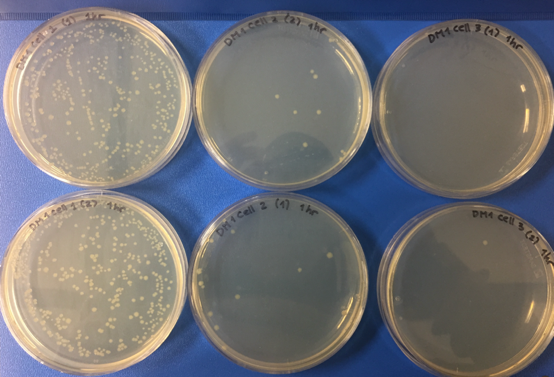


*E. coli* QCHI90/Cell 1h


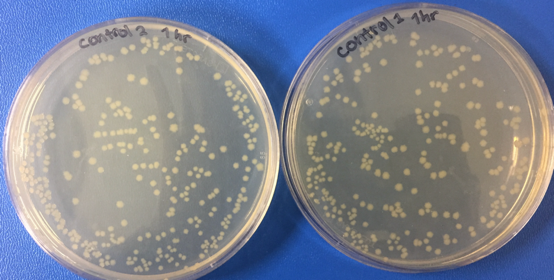


*E. coli* control 1h


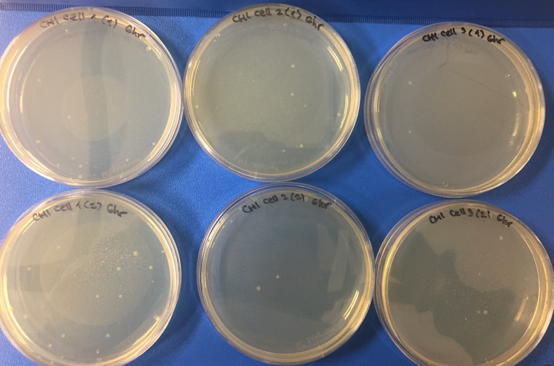


*E. coli* CHI/Cell 6h


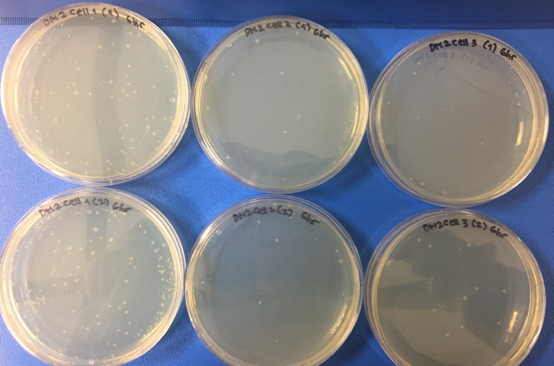


*E. coli* QCHI45/Cell 6h


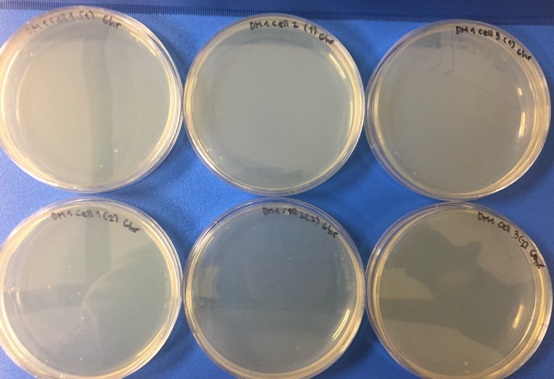


*E. coli* QCHI90/Cell 6h


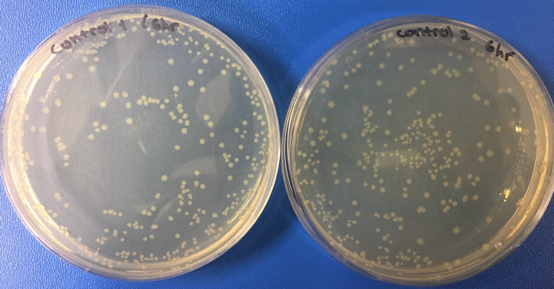


*E. coli* control 6h


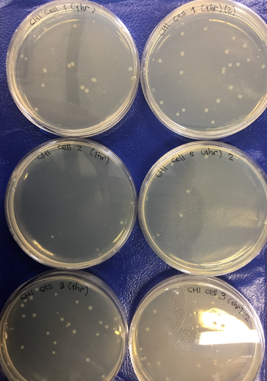


*B. subt.* CHI/Cell 1h


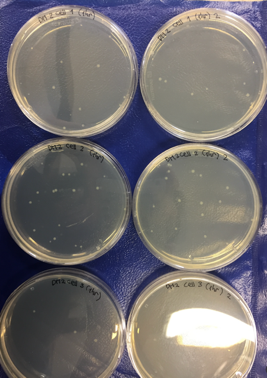


*B. subt.* QCHI45/Cell 1h


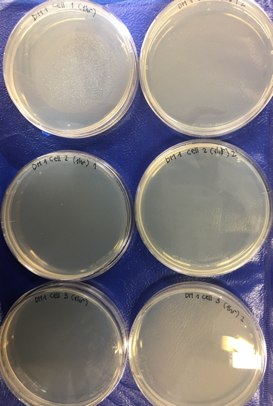


*B. subt.* QCHI90/Cell 1h


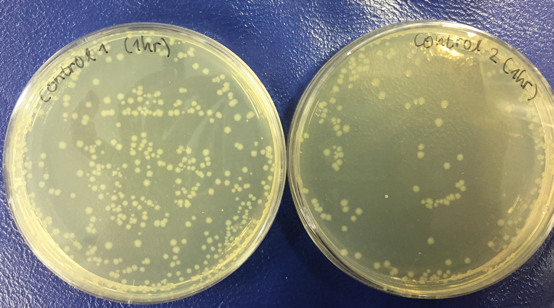


*B. subt.* control 1h


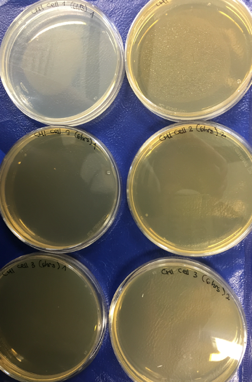


*B. subt.* CHI/Cell 6h


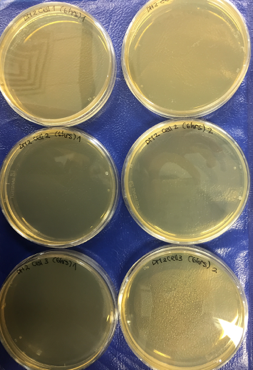


*B. subt.* QCHI45/Cell 6h


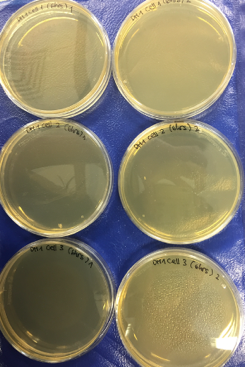


*B. subt.* QCHI90/Cell 6h


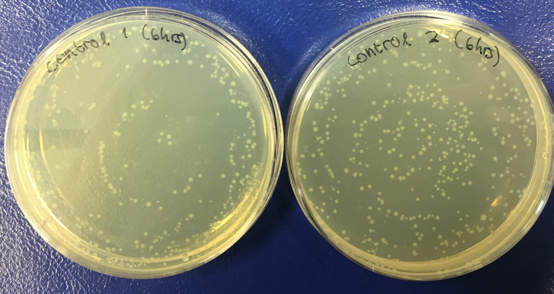


*B. subt.* control 6h

## References

1. Seong, H.-S., Whang, H. S. & Ko, S.-W. Synthesis of a quaternary ammonium derivative of chito-oligosaccharide as antimicrobial agent for cellulosic fibers. *J. Appl. Polym. Sci.* **76**, 2009–2015 (2000).

2. Lim, S.-H. & Hudson, S. M. Synthesis and antimicrobial activity of a water-soluble chitosan derivative with a fiber-reactive group. *Carbohydr. Res.* **339**, 313–319 (2004).
